# Knowledge, attitude, and practice of healthcare professionals regarding infection prevention at Gondar University referral hospital, northwest Ethiopia: a cross-sectional study

**DOI:** 10.1186/s13104-019-4605-5

**Published:** 2019-09-09

**Authors:** Teshiwal Deress Yazie, Gezahegn Bewket Sharew, Wondwossen Abebe

**Affiliations:** 1 Unit of Quality Assurance and Laboratory Management, School of Biomedical and Laboratory Sciences, College of Medicine and Health Sciences, University of Gondar, P. O. Box 196, Gondar, Ethiopia; 2 Immunology and Molecular Biology Department, School of Biomedical and Laboratory Sciences, College of Medicine and Health Sciences, University of Gondar, P. O. Box 196, Gondar, Ethiopia; 3 Microbiology Department, School of Biomedical and Laboratory Sciences, College of Medicine and Health Sciences, University of Gondar, P. O. Box 196, Gondar, Ethiopia

**Keywords:** Healthcare professional, Knowledge, Attitude, Practice, Safety, Ethiopia

## Abstract

**Objectives:**

Workplace health and safety is vital in every organization particularly in the healthcare settings. The aim was to assess the levels of knowledge, attitude, and practices of the healthcare professionals towards safety at Gondar University referral hospital. An institution based cross-sectional study was conducted from February to June 2018. Proportional random sampling technique was used to include 282 study participants and data were collected using a structured self-administered questionnaire and analyzed using SPSS version 20.

**Results:**

Among 282 study participants, 230 (81.6%), 181 (64.2%), and 162 (57.4%) had adequate knowledge, favorable attitude, and adequate practice scores, respectively. More than half (55.3%) of the study participants were untrained. There was a high (26.6%) prevalence of needlestick injury; however, the use of post-exposure prophylaxis after potential exposures was very limited. Generally, the levels of knowledge, attitude, and practice scores among the study participants were low. Therefore, there should be adequate and consistent supply of personal protective devices and other materials used for infection prevention and control. In addition, there should be awareness raising mechanism, including the provision of job aids and periodic training. Further, comprehensive studies should be conducted by including different types and levels of health facilities.

## Introduction

Workplace health and safety is a critical element in every organization particularly in the healthcare settings [1]. Healthcare professionals (HCPs) may act as a mechanical vector for the transmission of nosocomial infections from patient to patient [2, 3]. Accidental exposure to body fluids can cause bloodborne infections particularly hepatitis B virus (HBV), human immunodeficiency virus (HIV), and hepatitis C virus (HCV) [4]. Exposures may happen through needlestick or sharp injuries contaminated with infected body fluids or through contact with splashes [5]. The risk of seroconversion after percutaneous exposure to infected blood is approximately 0.1–0.3% for HIV, 2% for HCV, and 6–60% for HBV [4]. Safety practices help to protect healthcare workers, patients, and visitors from health hazards [6–8]. It includes the use of personal protective equipment (PPE) including gloves, gowns, eye goggles, aprons, masks [7], and through the provision of professional immunization programs [9]. With inadequate infection prevention practice, the risk of acquiring infections through exposure to body fluids is substantial [10].

Infection is one of the most important challenges in the healthcare facilities worldwide. It constituted the morbidity and mortality among the exposed groups [11]. All individuals particularly HCPs are potentially at high risk of infection due to their frequent exposure to body fluids [12–18]. Lack of proper protective devices and the absence of enabling working environment lead to poor compliance of safety precautions [19]. Hospital-acquired infections are the main problems associated with the healthcare services [20, 21] and currently, more than 1.4 million people are suffering from hospital-acquired infections worldwide and the risk is 2–20 times higher among the developing countries [22–24]. The World Health Organization (WHO) estimated that about 3 million healthcare workers are exposed to bloodborne viruses each year. Among this, about 90% of the infections were occurred in the developing countries [15].

The associated burden of disease related to hospital-acquired infections is prolonged hospital stay, long-term disability, increased microbial resistance, financial burden and deaths [3, 25, 26]. Although safety is an important component of the quality health services, currently there is limited information available regarding the level of knowledge, attitude and practice among the HCPs in the study area. Therefore, this study was aimed to assess the levels of knowledge, attitude, and practice scores of the HCPs towards safety at Gondar University referral hospital. The study will help policymakers in design and development of the appropriate infection prevention programs, and strategic plans.

## Main text

### Methods

#### Study design and setting

An institution based cross-sectional study was conducted from February to June 2018 at Gondar University referral hospital. The hospital is found in Gondar town at 748 km far from Addis Ababa, a capital city of Ethiopia, to the northwest. It is a multidisciplinary specialized teaching hospital with 550 inpatient beds. Currently, it provides health services to more than 5 million inhabitants in its catchment area [27]. According to the hospital human resource report, during 2018 there were 902 registered fulltime employee HCPs from which 86, 483, 95, and 110 were medical doctors, nurses, midwives, and medical laboratory professionals, respectively.

#### Sample size, sampling technique, and eligibility

The sample size was determined using a single population proportion formula. The final sample size was estimated to be 282 including 10% nonresponse rates. Proportional random sampling technique was used to include 282 study participants in the study. Only full-time employee HCPs who were potentially at high-risk (medical doctors, medical laboratory technologists, nurses, and midwives), available during the data collection period and consented to take part in the study were included.

#### Data collection and quality control

A structured self-administered questionnaire was used to collect the data. The questionnaire was developed after a review of the available scientific papers in the subject area [27–32]. Four main sections (socio-demographic characteristics, knowledge, attitude, and practice) were included in the data collection tool (Additional file 1). The tool was pretested on 10% of the similar study population and then the contents were slightly modified and suggestions from different experts were included. After training was provided, three medical laboratory professionals were involved in the data collection process and the medium of instruction was English. Information sheet and consent forms were included in the first part of the questionnaire. The questionnaire was distributed to the study participants in the form of hard copies. Then study participants were requested to read the informed-consent statements before they proceed to the next section. Study participants who unwilling to take part in the study were asked to return questionnaires to the data collectors. The questionnaires were collected, checked for completeness and any incomplete questionnaire was taken back to the respondent for completion. In addition, timely supervision of the data collection process was done by the investigators. Further, the data were tested statistically for internal consistency (reliability) using Cronbach’s alpha test. The overall Cronbach’s alpha value was 0.695 which indicates that the items are somewhat inter-related.

#### Methods of measurement and analysis

Data were coded and entered into Epi info version 3.5.1 software and exported into SPSS version 20 for analysis. Knowledge and practice questions were scored as 1 or 0 for correct and incorrect responses, respectively. Whereas, attitude responses were provided 1, 2, or 3 for “Disagree”, “Neutral” and “Agree”, respectively. Summary statistics such as frequencies and proportions were computed as appropriate. Mean scores were calculated and used as a cut point to dichotomize the outcome variables. Scores below the mean scores were considered as inadequate knowledge, unfavorable attitude, or inadequate practice; whereas, scores equal to the mean score or above were considered as adequate knowledge, favorable attitude, or adequate practice. The cut-off value for the knowledge, attitude, and practice scores were adapted from similar studies [33–36].

#### Ethical considerations

Ethical clearance and support letters were obtained from the Ethical Review Committee (ERC) of the School of Biomedical and Laboratory Sciences, University of Gondar. The support letter was submitted to the Gondar University referral hospital. Then, permission was obtained from the hospital director and department/section heads. Study participants were informed about the purpose and importance of the study through written informed concent before the data collection process. In addition, participants who were unwilling to take part in the study and those who need to quit their participation at any stage were informed to do so without any restriction.

### Results

#### Socio-demographic characteristics

Among 282 study participants, 143 (50.7%) were male and the age was ranged from 23 to 53 years. Concerning their job categories, 176 (62.4%), 31 (11%), 40 (14.2%), and 35 (12.4%) were nurses, doctors, laboratorians, and midwives, respectively. About 73 (25.9%), 62 (22%), and 147 (52.1%) had ≤ 2 years, 3–4 years, and ≥ 5 years of work experience, respectively. Most study participants (83.7%) were first-degree holders. Only 126 (44.7%) of them were taken training regarding infection prevention and safety. The majority (86.5%) of the participants were vaccinated for HBV (Table 1).

**Table 1:** Showing socio-demographic and health facility related characteristics for the evaluation of safety precaution in Gondar University referral hospital, Northwest Ethiopia, 2018

Socio-demographic and HC related variables	Variable category	Frequency n (%)
Age group	≤ 25 years	26 (9.2%)
	26–30 years	166 (58.9%)
	≥ 31 years	90 (31.9%)
Working department	OPD	60 (21.3%)
	Ward	139 (49.3%)
	Laboratory	32 (11.3%)
	Emergency	24 (8.5%)
	Others	27 (9.6%)
Working hours per day	8 h	180 (63.8%)
	More than 8 h	102 (36.2%)
Number of study participants by the dose of HBV vaccine they completed	First dose	12 (4.3%)
	Second dose	40 (14.2%)
	Third dose	190 (67.4%)
Availability of sufficient quantity PPE in the department	Yes	118 (41.8%)
	No	139 (49.3%)
	Not sure	25 (8.9%)
Availability of safety guidelines/manuals in the department/section	Yes	135 (47.9%)
	No	118 (41.8%)
	Not sure	29 (10.3%)
Source of information about safety precaution	Training	57 (20.2%)
	Guideline	129 (45.7%)
	Friend	63 (22.3%)
	Others	33 (11.7%)

*HC* health care, *HBV* hepatitis B virus, *PPE* personal protective equipment, *BSc* Bachelor of Science, *MSc* Master of Science, *OPD* outpatient department

#### Knowledge of study participants

In this study, the mean knowledge score of the study participants was 10.69. Among 282 study participants, 230 (81.6%) of them had adequate knowledge score. About 230 (81.6%) of them correctly identified that a safety box should be filled only a maximum of three-fourth. Similarly, 231 (81.9%) of the study participants knew that 72 is the maximum time delay to start HIV post-exposure prophylaxis. Additional findings on the knowledge scores is attached as an Additional file 2.

#### Attitude of study participants

The mean attitude score of the study participants was 9.65. Based on this cutoff value 181 (64.2%), and 101 (35.8) of the study participants had favorable and unfavorable attitude scores, respectively. Almost all the study participants 280 (99.3%) respond that safety precaution is important for the healthcare facilities. Similarly, 272 (96.5%) of the study participants believed that healthcare workers are potentially at risk of infection (Table 2).

**Table 2:** Frequency of study participants among each Likert type of variables at Gondar University referral hospital, Northwest Ethiopia, 2018

Variables	Response		
	Agree n (%)	Disagree n (%)	Neutral n (%)
Occupational safety training is important for health professionals	274 (97.2)	6 (2.1)	2 (0.7)
Healthcare environments can expose HCPs to a health hazard	260 (92.2)	15 (5.3)	7 (2.5)
HCPs workplace related risk exposure is a major crisis	213 (75.5)	42 (14.9)	27 (9.6)
Workplace risk assessment is important for occupational safety	279 (98.9)	2 (0.7)	1 (0.4)
Sharp materials should be discarded in a safety box	278 (98.6)	3 (1.1)	1 (0.4)
Needles should be recapped before disposal	103 (36.5)	167 (59.2)	12 (4.3)
Wearing PPE during the healthcare delivery process is mandatory	251 (89)	23 (8.2)	8 (2.8)
Vaccination of healthcare workers is necessary	263 (93.3)	17 (6.0)	2 (0.7)
HBV can be transmitted through biomedical wastes	248 (93.3)	17 (6.0)	2 (0.7)

#### Practice of study participants

The mean and adequate practice scores of the study participants were 7.65 and 162 (57.4%), respectively. Regarding previous exposure to biohazards, about one-fourth (26.6%) of the study participants were encountered needlestick/sharps injuries during the previous 12 months preceding the data collection period. Among them, only 49 (17.4%) had taken HIV post-exposure prophylaxis. Regarding mobile phone usage during their professional practice, about one-third (33%) of the study participants responded that they have always answered their phones with glove hands. With respect to segregation of healthcare wastes, 219 (77.7%) of them segregated according to their type (Table 3).

**Table 3:** Frequency of the study participants among safety practice-related variables at Gondar University referral hospital, Northwest Ethiopia, 2018

Variables	Response		
	Always n (%)	Sometimes n (%)	Not at all n (%)
Use safety guideline/manual at the workplace	67 (23.8)	143 (50.7)	72 (25.5)
Wear gloves during risky procedures	280 (88.7)	32 (11.3)	0
Wash hands with detergent after contact with patients	155 (55)	122 (43.3)	5 (1.8)
Use of PPE during professional practice	149 (52.8)	122 (44.3)	8 (2.8)
Clean working environment at the end of the working time	138 (48.9)	130 (46.1)	14 (5)
Monitor working environment waste management system	116 (41.1)	135 (47.9)	13 (4.6)
Changing gloves between patients	182 (64.5)	89 (31.6)	11 (3.9)
Wash hands after removal of gloves	146 (51.8)	131 (46.5)	5 (1.8)
Recap used needles before disposal	101 (35.8)	54 (19.8)	127 (45)
Treat infectious wastes with appropriate disinfectants	180 (63.8)	73 (25.9)	29 (10.3)

*PPE* personal protective equipment

### Discussion

Safety is an important component of every healthcare organization which enables to provide quality health services and essential to protect health workers, patients, and community from health-related risks. Particularly, exposure to body fluids containing infectious agents has long been recognized as a potential threat to the HCPs. The current study showed that 81.6% of the study participants had adequate knowledge score regarding safety precautions. This finding is more or less comparable to 84% and 84.7% findings from the health institutions of Bahir Dar and Debre Markos town, respectively [37, 38]. This result was better than 50% a finding from Nigeria [39]. The possible explanation for the difference could be due to a difference in methodology, educational level, work experience, training opportunity, and personal characteristics of the study participants between the countries.

When each of the specific components of safety precautions was analyzed, better results have been obtained in some items. For instance, a relatively high proportion of HCPs have awareness on the transmission mechanisms of infectious agents (98.6%), proper handling of used needles and sharps (98.9%), and importance of wearing PPE during the clinical practices (95.7%). On the other hand, about 81.6% of study participants aware that only a maximum three-fourth of the safety box should be filed to prevent needle stick injuries which were quite unsatisfactory. Regarding HCPs awareness on the potential risks of their working environment, 89% of them aware that they were working in a risk full environment which is relatively quite satisfactory. On the other hand, only 77.3% of them aware of how to perform work environment risk assessment. With respect to the segregation of medical wastes, only 81.6% of them had awareness of separation of wastes according to their type before disposal. This means that about 18% HCPs may probably dispose of wastes randomly in any waste container and this malpractice may increase the amount of biologically hazardous waste generated from the healthcare facilities. This is because when the infectious waste type is mixed with the general waste type then the entire mass will be unnecessarily polluted.

With respect to study participants’ attitude, unacceptably low (64.2%) number of study participants had favorable attitude score regarding safety precautions. Concerning the PPE utilization, 89% of the study participants believe that using the PPE while accidents happen during the healthcare delivery procedure is mandatory. This finding is slightly better than a study from the governmental healthcare facilities of Addis Ababa (80%) [40]. This difference might be attributed to the difference in the academic background of the study respondents, sampling technique and sample size between studies.

In the current study, an unacceptably low (57.4%) proportion of the study participants had adequate practice score regarding safety precaution which is lower than a study conducted among the government healthcare facilities of Addis Ababa (66.1%) [40]. The result is; however, more or less comparable to 54.2% and 60.5% studies from Bahir Dar and Wolaitta, respectively [31, 37]. Hand washing practice after patient contact/handling of infectious wastes is the most important safety precaution practice; however, in this study, only (55%) of the HCPs studied were complied hand washing practice after patient contact/handle biologically hazardous substances. This finding is lower than a study from public health facilities of Mekelle in which about 61.5% of study participants always washed their hands [41]. With respect to injuries related to needle stick injuries, a year prevalence rate of needlestick injury (26.6%) in this study was unacceptably high. Better findings 17.5% and 18.7% were obtained from different parts of Ethiopia [42, 43]. This result is; however, much lower than a study conducted among the governmental healthcare facilities of Addis Ababa (30%) [40]. In this study changing gloves between patient contacts was 64.5% which is far lower than the previous study in Gondar university hospital (80.6%) [27]. The practice of HCPs related to wearing gloves during risky procedures about 88.7% of the study participants, this study the is similar to the previous the studies in this area (88.7%) [27].

### Conclusions

Generally, the levels of knowledge, attitude, and practice scores among the HCPs were found to be low and there was a high prevalence of needlestick/sharp injuries. However, the use of PEP after potential exposures was very limited. Therefore, the hospital should ensure adequate and consistent supply of personal protective devices and other materials used for infection prevention and control. In addition, there should be awareness raising mechanism, including the provision of job aids and periodic training on proper hand washing, PEP administration protocols, and safe disposal of biologically hazardous wastes including evaluation of the trainees’ practices through observation by trainers to assess their level of competency against minimum acceptable standards. In addition, HCPs should be committed to safety practices. Further, periodic and comprehensive studies should be conducted by including different types and levels of health facilities on a large scale.

## Limitations

Data obtained from the study participants through self-report were not cross-checked with their actual practices on the ground. Further, since the study was conducted in a single health facility, it could not be generalized at a national level.

## Supplementary information

**Additional file 1.** Data collection tool.

**Additional file 2: Table S1.** Knowledge of the study participants about safety precaution at Gondar University referral hospital, Northwest Ethiopia, 2018.

## Data Availability

The data collection tool used in this study to collect data on knowledge, attitude, and practices among the HCPs is attached as an additional file.

## Abbreviations

HCPs: healthcare professionals
HBV: hepatitis B virus
HCV: hepatitis C virus
HIV: human immune deficiency virus
PPE: personal protective equipment
SPSS: Statistical Package for Social Sciences
