# Supplementary material for: Knowledge, attitude, and practice of healthcare professionals regarding infection prevention at Gondar University referral hospital, northwest Ethiopia: a cross-sectional study
Source: BMC Res Notes. 2019 Sep 9;12:563. doi: 10.1186/s13104-019-4605-5 (PMC6734428; doi:10.1186/s13104-019-4605-5)
Supplement: Supplementary file 1 — Additional file 1. Data collection tool. [file 13104_2019_4605_MOESM1_ESM.docx]

**University of Gondar**

**College of Medicine and Health Sciences and referral hospital**

**School of Biomedical and Laboratory Sciences**

**Information sheet**

***Title of the study:*** Assessment of knowledge, attitude, and practice of safety among healthcare professionals at Gondar University referral hospital, Northwest Ethiopia

***Background:*** Workplace health and safety is a critical element in every healthcare organization. Healthcare professionals can act as a vehicle for the transmission of nosocomial infections.

***Objective:*** To assess knowledge, attitude, and practice of safety among healthcare professionals at Gondar University referral hospital, northwest Ethiopia

***Perceived benefits and risk:*** The result may healthcare professionals, healthcare facility managers, researchers, policymakers and other stakeholders as appropriate. Being involved in this study does not induce any risk you will face.

***Participation and withdrawal****:* Your participation which will take you about 8 minutes is fully voluntarily. You will be free to withdraw from the study at any time or not to answer questions if you want to do so.

***Confidentiality:*** All personal identifiers and information will not be taken hence your responses will be kept confidential. Data will be accessed by the principal investigator, advisors and research assistant only and finally will be analyzed anonymously.

***Persons to contact:*** If you have, questions/ concerns about this study you can contact;

Teshiwal Deress: (Email: [teshiwalderess@gmail.com](mailto:teshiwalderess@gmail.com))

Gezahegn Bewket: (Email: [gezahegnb123@gmail.com](mailto:gezahegnb123@gmail.com))

Wondwosen Abebe: (Email: [wondisweet@gmail.com](mailto:wondisweet@gmail.com))

**Consent form**

Dear participant! you are among the study participants selected from the healthcare facility. It is your full right to participate in this study; however, your honest participation will help us to get important data on knowledge, attitude, and practice of safety precautions and associated factors, so, you are kindly requested to give your honest responses and keep participation. Would you be willing to participate, please?

1. Yes 2. No

Dear interviewer! For the sake of confidentiality please do not write participants name or another personal identifier on the questionnaire!

**Part I. Socio-Demographic and health care facility related characteristics**

Dear participant, the following questions are targeted to differentiate your socio-demographic characteristics. Please circle the best proper choice of answer code.

| **No** | **Question** | **Answer options and codes** | **Remark** |
| --- | --- | --- | --- |
|  | Gender | 1. Male 2. Female |  |
|  | Age in years | __________years |  |
|  | Religion | 1. Orthodox 2. Muslim 3. Protestant 4. Catholic 5. Others (specify) |  |
|  | Marital status | 1. Single 2. Married 3. Divorced 4. Widowed |  |
|  | Profession | 1. Medical doctor 2. Medical laboratory 3. Midwifery 4. Nurse |  |
|  | Educational level | 1. Diploma 2. First degree 3. Second degree and above |  |
|  | Working department/section | 1. OPD 2. Ward 3. Laboratory room 4. Emergency 5. Others (specify) |  |
|  | Work experience in years | __________years |  |
|  | Working hours per day | ___________hours |  |
|  | Have you taken risk assessment training before? | 1. Yes 2. No |  |
|  | Have you taken the HBV vaccine? | 1. Yes 2. No | **If no, skip to**  **Qn 113** |
|  | If your answer on Qn 111 is yes, how many doses have taken? | 1. The first dose only 2. The second dose only 3. Third dose (complete) |  |
|  | Are there sufficient personal protective devices available in your department/ section? | 1. Yes 2. No 3. I am not sure |  |
|  | Are safety manuals/ guidelines available in your department/ section? | 1. Yes 2. No 3. I am not sure |  |
|  | Where did you get information about safety precaution? (you can encircle more than one options) | 1. From training 2. From guidelines, books, articles... 3. From friend 4. From other sources |  |

**Part II. Knowledge questions**

Dear participants, the following questions are the target to see your knowledge of safety precautions. Please circle the best choice of your answer code.

| **No** | **Questions** | **Answer options and codes** |
| --- | --- | --- |
|  | Is occupational safety a problem for healthcare organizations? | 1. Yes 2. No |
|  | Are healthcare workers responsible for occupational health and safety? | 1. Yes 2. No |
|  | Do you know how to use personal protective equipment? | 1. Yes 2. No |
|  | Do you know how to perform a risk assessment? | 1. Yes 2. No |
|  | Do you know transmission mechanisms of infectious agents? | 1. Yes 2. No |
|  | Do you wash your hands before and after you contact with patents? | 1. Yes 2. No |
|  | Are you aware of the risks of your working environment? | 1. Yes 2. No |
|  | Do you know how to handle used needles and sharps safely? | 1. Yes 2. No |
|  | Do you know about color coding segregation of healthcare wastes? | 1. Yes 2. No |
|  | How maximum full should be the safety box containing sharp medical supplies? | 1. ^1^/2 full 2. 3/4 full 3. Full 4. I don't know |
|  | According to the World Health Organization guideline, what is the maximum delay to start HIV post-exposure prophylaxis? | 1. 24 hours 2. 48 hours 3. 72 hours 4. I don't know |
|  | Is there any health hazard associated with healthcare wastes? | 1. Yes 2. No 3. I don't know |
|  | Does wearing personal protective equipment reduce the risk of infection? | 1. Yes 2. No 3. I don't know |

**Part III: Attitude questions**

Dear participant, the following questions are targeted to see your attitudes towards safety precautions. Please circle the best choice of your answer code.

| **No** | **Question** | **Answer options and codes** |
| --- | --- | --- |
|  | Safety precaution is important for healthcare organizations | 1. Agree 2. Disagree 3. Neutral |
|  | Occupational health and safety training is important for healthcare workers | 1. Agree 2. Disagree 3. Neutral |
|  | Your healthcare environment may expose you to occupational hazards | 1. Agree 2. Disagree 3. Neutral |
|  | Health care workers are at high risk of infection | 1. Agree 2. Disagree 3. Neutral |
|  | All personal protective equipment should be accessible in the working department/ section of the healthcare facility. | 1. Agree 2. Disagree 3. Neutral |
|  | Individual workplace risk exposure should be considered as a crisis of community | 1. Agree 2. Disagree 3. Neutral |
|  | Risk assessment is important for occupational health and safety. | 1. Agree 2. Disagree 3. Neutral |
|  | Sharp materials should be discarded in a safety box | 1. Agree 2. Disagree 3. Neutral |
|  | Needles should be re recapped after use | 1. Agree 2. Disagree 3. Neutral |
|  | If you didn't have taken HBV vaccine before, are you willing to take it? | 1. Agree 2. Disagree 3. Neutral |
|  | Wearing facemask and eye goggles during procedures with aerosol production is mandatory | 1. Agree 2. Disagree 3. Neutral |
|  | Vaccination for healthcare workers is mandatory | 1. Agree 2. Disagree 3. Neutral |
|  | Hepatitis B virus may be transmitted through biomedical wastes | 1. Agree 2. Disagree 3. Neutral |
|  | Is there any safety/ infection prevention guideline/ document in your department? | 1. Yes 2. No 3. I don't know |

**Part IV Practice questions**

Dear participants, the following questions are the target to see your practices towards safety precautions. Please circle the best choice of your answer code.

| **No.** | **Questions** | **Answer options and codes** | **Remark** |
| --- | --- | --- | --- |
|  | Have you ever encountered any sharp /needlestick injury in the last 12 months? | 1. Yes 2. No | **If no, skip to Qn 403** |
|  | If yes, on question No 401 have you taken prophylaxis for HIV? | 1. Yes 2. No |  |
|  | Did you answer your cell phone with the glove while calling before? | 1. Yes 2. No |  |
|  | How often do you use safety guideline/ manual at your workplace? | 1. Always 2. Sometimes 3. Not at all |  |
|  | How often do you wear gloves during risky procedures? | 1. Always 2. Sometimes 3. Not at all |  |
|  | How often do you wash your hands with proper detergent after contact with patients/ working time? | 1. Always 2. Sometimes 3. Not at all |  |
|  | How often do you use proper personal protective equipment during your professional practice? | 1. Always 2. Sometimes 3. Not at all |  |
|  | How often do you clean your working area after the end of working shift? | 1. Always 2. Sometimes 3. Not at all |  |
|  | How often do you monitor your working area waste management system? | 1. Always 2. Sometimes 3. Not at all |  |
|  | How often do you practice separate disposal of healthcare wastes? | 1. Always 2. Sometimes 3. Not at all |  |
|  | How often do you perform risk assessment in your working department/ section? | 1. Always 2. Sometimes 3. Not at all |  |
|  | How often do you change gloves between contacts with different patients? | 1. Always 2. Sometimes 3. Not at all |  |
|  | How often do wash your hands after removal of gloves? | 1. Always 2. Sometimes 3. Not at all |  |
|  | How often do you recap used needles? | 1. Always 2. Sometimes 3. Never |  |
|  | Do you segregate healthcare wastes according to their type at the point of generation? | 1. Yes 2. No |  |
|  | How often do you treat infectious wastes with disinfectants? | 1. Always 2. Sometimes 3. Not at all |  |
