# Supplementary material for: Knowledge, attitude, and practice of healthcare professionals regarding infection prevention at Gondar University referral hospital, northwest Ethiopia: a cross-sectional study
Source: BMC Res Notes. 2019 Sep 9;12:563. doi: 10.1186/s13104-019-4605-5 (PMC6734428; doi:10.1186/s13104-019-4605-5)
Supplement: Supplementary file 2 — Additional file 2: Table S1. Knowledge of the study participants about safety precaution at Gondar University referral hospital, Northwest Ethiopia, 2018. [file 13104_2019_4605_MOESM2_ESM.docx]

**Table S1** Knowledge of the study participants about safety precaution at Gondar University referral hospital, Northwest Ethiopia, 2018.

| **Variables** | **Responses** | |
| --- | --- | --- |
|  | **Yes (%)** | **No (%)** |
| Awareness of whether occupational safety is a concern for the healthcare facility | 247 (87.6) | 35 (12.4) |
| Healthcare workers’ responsibility for occupational health and safety | 270 (95.7) | 12 (4.3) |
| Perception of how to effectively use personal protective equipment | 275 (97.5) | 7 (2.5) |
| Awareness of how to perform a risk assessment | 218 (77.3) | 64 (22.7) |
| Awareness transmission mechanisms of infectious agents | 278 (98.6) | 4 (1.4) |
| Awareness of the potential risks of the working environment | 251 (89.0) | 31 (11.0) |
| Awareness on how to handling of used needles and sharps | 279 (98.9) | 3 (1.1.0) |
| Perception of color-coding segregation of healthcare wastes | 230 (81.6) | 51 (18.1) |
| Awareness of the potential health hazard associated with medical wastes | 236 (83.7) | 46 (16.3) |
| Awareness regarding the importance of wearing personal protective equipment | 270 (95.7) | 12 (4.3) |
